# Aberrant Glycosylation of Anchor-Optimized MUC1 Peptides Can Enhance Antigen Binding Affinity and Reverse Tolerance to Cytotoxic T Lymphocytes

**DOI:** 10.3390/biom6030031

**Published:** 2016-06-29

**Authors:** Latha B. Pathangey, Vani Lakshminarayanan, Vera J. Suman, Barbara A. Pockaj, Pinku Mukherjee, Sandra J. Gendler

**Affiliations:** 1 Department of Biochemistry and Molecular Biology, College of Medicine, Mayo Clinic in Arizona, 13400 E. Shea Boulevard, Scottsdale, AZ 85259, USA; pathangey.latha@mayo.edu (L.B.P.); lakshminarayanan.vani@gmail.com (V.L.); 2 Department of Immunology, College of Medicine, Mayo Clinic in Arizona, 13400 E. Shea Boulevard, Scottsdale, AZ 85259, USA; pmukherj@uncc.edu; 3 Cancer Center Statistics, Mayo Clinic, 200 First Street SW, Rochester, MN 55905, USA; suman.vera@mayo.edu; 4 Department of Surgery, Mayo Clinic in Arizona, Mayo Clinic Hospital, 5777 E. Mayo Boulevard, Phoenix, AZ 85054, USA; pockaj.barbara@mayo.edu

**Keywords:** Tn antigen, vaccine, immunotherapy, CTL, breast cancer, Mucin1, heteroclitic antigen

## Abstract

Cancer vaccines have often failed to live up to their promise, although recent results with checkpoint inhibitors are reviving hopes that they will soon fulfill their promise. Although mutation-specific vaccines are under development, there is still high interest in an off-the-shelf vaccine to a ubiquitous antigen, such as MUC1, which is aberrantly expressed on most solid and many hematological tumors, including more than 90% of breast carcinomas. Clinical trials for MUC1 have shown variable success, likely because of immunological tolerance to a self-antigen and to poor immunogenicity of tandem repeat peptides. We hypothesized that MUC1 peptides could be optimized, relying on heteroclitic optimizations of potential anchor amino acids with and without tumor-specific glycosylation of the peptides. We have identified novel MUC1 class I peptides that bind to HLA-A*0201 molecules with significantly higher affinity and function than the native MUC1 peptides. These peptides elicited CTLs from normal donors, as well as breast cancer patients, which were highly effective in killing MUC1-expressing MCF-7 breast cancer cells. Each peptide elicited lytic responses in greater than 6/8 of normal individuals and 3/3 breast cancer patients. The CTLs generated against the glycosylated-anchor modified peptides cross reacted with the native MUC1 peptide, STAPPVHNV, suggesting these analog peptides may offer substantial improvement in the design of epitope-based vaccines.

## 1. Introduction

It has been difficult to develop effective vaccines for cancer, as the cancer cells are largely similar to normal cells and our immune system avoids attacking itself. Tumors develop over a long period of time and lack the strong inflammatory response that bacteria and viruses elicit, which activates a strong immune response. The end result is that immune cells tolerate tumor growth. Over time, tumors develop many resistance mechanisms that result in a microenvironment that suppresses a functional immune system. Thus, cancer vaccines may be made more effective if combined with something that overcomes the immunosuppressive environment of the tumor.

Cancer immunotherapy is undergoing a revolution with the emergence of checkpoint inhibitors such as anti-PD-1 and anti-CTLA4. These antibodies strengthen natural immune responses against cancer by blocking the triggering of inhibitory receptors on activated T cells, resulting in major clinical responses in up to 40%–60% of patients with advanced melanoma [1]. Subsets of patients with several additional types of cancer have also responded dramatically to checkpoint blockade, including bladder, kidney, non-small cell lung cancer and Hodgkin’s disease [1]. Unfortunately, other types of cancer have not shown responses, in particular, most breast and pancreatic cancer, which are immune quiescent tumors [2,3,4], although small numbers of patients with triple negative breast cancer have been reported as responsive [5]. The checkpoint inhibitors appear to work most effectively in immune-driven cancers or those with a high mutational load [6,7]. It is likely that tumors fortified with T cells that recognize their tumor may be rendered more responsive to checkpoint inhibitor therapy [3,8].

MUC1 is a tumor-associated protein expressed by most solid tumors, including more than 90% of breast and pancreas tumors as well as multiple myelomas and lymphomas; even early stage triple negative breast tumors show almost uniform expression of this antigen [9], although others have shown that MUC1 expression is heterogeneous and reduced in clinically progressive triple negative breast cancers compared with luminal breast cancers [10]. MUC1 was recently ranked by a National Cancer Institute working group as one of the two most promising cancer vaccine target antigens for clinical development, based on therapeutic function, immunogenicity, role in oncogenicity, expression level and percent of antigen-positive cells, stem cell expression, number of patients with antigen-positive cancers, number of antigenic epitopes, and cellular location of antigen expression [11]. Cancer-derived MUC1 stimulates both humoral and cellular immunity, its normal apical distribution is lost in cancer cells and aberrant glycosylation exposes peptide epitopes and novel carbohydrate antigens such as Thompson-Friedenreich (TF: Gal(1-3)-α-GalNAc-*O*-serine/threonine) and Tn, the monosaccharide precursor of TF (α-GalNAc-*O*-serine/threonine)), which are not found in normal tissues [12,13,14]. Tn is found in most breast tumors and appears early during the tumorigenic process [14], making it an excellent example of tumor-specific glycosylation.

These observations have led to the development of a number of clinical trials with MUC1 vaccines, with variable success. Therapeutic effects employing both glycosylated and non-glycosylated MUC1 vaccines have been observed. The MUC1 tandem repeat non-glycosylated, lipid-encapsulated peptide (BLP25, Tecemotide) administered with chemotherapy to regionally advanced non-small cell lung cancer patients elicited a 10-month survival advantage in an 806 patient subset [15]. A phase I trial of ONT-10, which consists of two tandem repeats aberrantly glycosylated with Tn and TF, led to disease stabilization in 65% of patients with advanced disease of multiple tumor types [16]. Disease stabilization was also seen in multiple myeloma patients treated with the MUC1 signal peptide [17] and in pancreatic cancer patients treated with dendritic cells pulsed with a 100-mer peptide [18]. Full length MUC1 has also been used as a vaccine. A recombinant virus Vaccinia Ankara encoding both MUC1 and IL-2, the TG4010 vaccine, together with chemotherapy in non-small cell lung cancer showed activity [19]. In a 15 year follow-up of a pilot Phase III trial of Stage II breast cancer patients treated with oxidized mannan linked to MUC1 with 5 TR, the recurrence rate was greatly reduced compared to placebo (two out of 16 patients versus nine out of 15 patients) [20].

Lack of durable responses may be due to the presentation of self-antigens to the immune system as tumors develop, leading to tolerance [21]. To break tolerance to self-antigens, it has proven beneficial to optimize anchor residues in peptide vaccines, which increases the major histocompatibility complex (MHC) binding affinity [22,23,24,25,26]. This likely increases the stabilization of the peptide-MHC complex, resulting in secretion of cytokines such as interferon gamma (IFNγ) and better immunogenicity [27,28,29].

Another approach for breaking tolerance involves immunization with peptides glycosylated with tumor-specific carbohydrates that are not found in normal tissues, which may result in heightened immunity as patients are less likely to be tolerant to these epitopes [30]. Many glycopeptide vaccine constructs have been designed, most often conjugating the Tn carbohydrate to MUC1 tandem repeat peptides and examining the immune response in mice. However, most immunological analyses in mice have been restricted to determining antibody production. We recently reported that glycopeptides (self-adjuvanting or not) can induce effective anti-tumor T cell responses in mouse models [31,32,33,34], suggesting that glycosylated vaccines have great potential. Tumor-specific glycopeptide vaccines may by-pass the problem of tolerance to self-proteins that has previously resulted in low-level responses to immunotherapy.

The objective of this study was to improve the design of a MUC1 vaccine for HLA-A*0201 individuals, relying on heteroclitic optimizations of potential anchor amino acids with and without tumor-specific glycosylation of the peptides. It is well known that the tandem repeat domain of MUC1 is poorly immunogenic and lacks optimal anchor amino acids [35]. Anchor peptide optimization and aberrant (Tn) glycosylation of the P1:STAPPVHNV MUC1 degenerate tandem repeat peptide resulted in high affinity binding to T2 cells expressing HLA-A*0201. Cytotoxic T cells (CTLs) were generated from bloods from normal post-menopausal HLA-A*0201women and breast cancer patients stimulated in vitro with allogeneic dendritic cells (DCs) pulsed with glycosylated and/or anchor-modified MUC1 peptides. The CTLs lysed MCF-7 breast cancer cells (MUC1^+^, HLA-A*0201), produced IFNγ, and showed cross-reactivity with the native P1:STAPPVHNV peptide, suggesting these analog peptides may offer substantial improvements in the design of epitope-based vaccines. Vaccination with optimal peptides combined with checkpoint inhibitors, such as anti-PD-1 or anti-CTLA-4, to overcome immune evasion may achieve high immune response rates and properly polarized T cell immune responses.

## 2. Results

### 2.1. Identification of Heteroclitic and Aberrantly Glycosylated Peptides for a Human Vaccine

Peptides that are presented by the HLA-A*0201 molecule are most often studied as this allele is expressed by forty-five percent of the human population and the peptide binding motifs are well-characterized [35,36,37]. Binding to HLA-A*0201 is optimal if there is a methionine or a leucine at position 2 and a valine or a leucine at position 9 [38]. Previous studies suggested glycosylation of the threonine at the fifth position may be ideal for GalNAc (Tn) conjugation, as there is evidence for a small cavity in the middle region of the TCR binding pocket into which a small sugar moiety may fit [30,36]. MUC1-derived peptides that were anchor-optimized with a leucine in the second position and/or glycosylated in the fifth position with aberrant glycosylation, Tn, were generated. The fifth proline in the P1:STAPPVHNV native peptide was changed to a threonine to allow for glycosylation in the fifth position. The last position was already an optimal valine. Peptides were chosen from known HLA-A2*0201 binders (Table 1).

### 2.2. Glycosylated and/or Anchor-Optimized Peptides Show Increased Stabilization of HLA-A*0201 Molecules on T2 Cells

**Figure 1:**
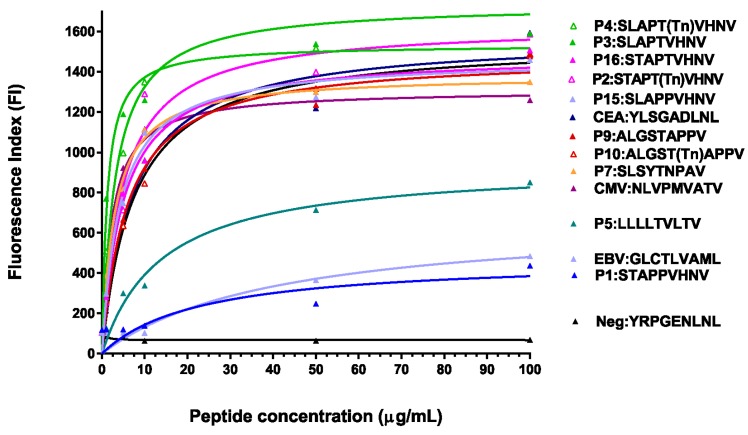
MUC1 peptides (anchor-optimized and glycosylated) stabilized HLA-A*0201 molecules on T2 cells. Increasing amounts of the MUC1 peptides (0–100 μg/mL) and β2-microglobulin (1 μg/mL) were incubated with T2 cells for 18 h at 37 °C prior to staining with the HLA-A*0201 antibody (clone BB7.2, Pharmingen). Mean fluorescence intensity (MFI) was determined by flow cytometry and the fluorescence index (FI) was calculated from the formula: FI = (F_S_ − F_B_)/(F_T2_ − F_B_) × 100 where F_S_ is the MFI of the test peptides, F_B_ is the non-peptide isotype antibody-stained control MFI, and F_T2_ is the non-peptide HLA-A*0201 antibody-stained control MFI.

Using fluorescence index (FI) as the measure of binding to the T2 cells expressing HLA-A*0201, we observed that the two previously described MUC1 peptides (P5:LLLLTVLTV (M1.2) and P1:STAPPVHNV (M1.1)) [39] bound moderately to the HLA-A*0201 molecule, whereas all modified MUC1 peptides had significantly increased binding to the HLA-A*0201 molecule on the T2 cells, with the binding being as good as or better than the CMV positive high binding control peptide (Figure 1). The data suggest there is increased stabilization of the glycosylated and/or anchor-modified peptides to the MHC class I/β2-microglobulin complex.

### 2.3. Peptide Modifications Increased Binding Affinities for HLA-A*0201 Molecules

**Figure 2:**
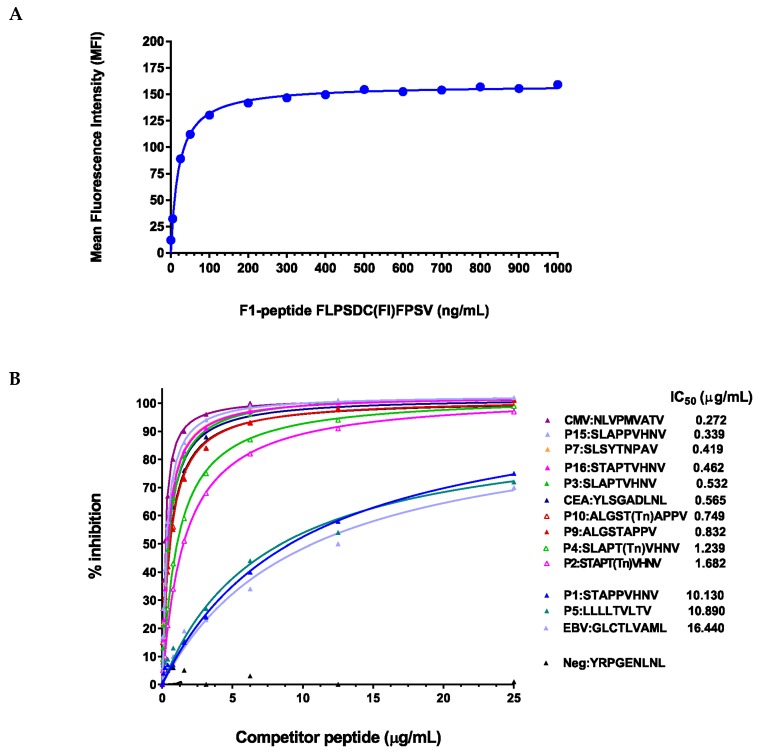
Peptide modifications increased binding affinities for HLA-A*0201 molecules (**A**) Titration of the reference peptide. The Hepatitis B core antigen (residues 18–27), an HLA-A*0201-binding peptide (FLPSDFFPSV), was synthesized with a cysteine residue substituted for the F (FLPSDCFPSV). This cysteine was conjugated to fluorescein (F1-peptide) for use in competitive inhibition studies. Recombinant β2-microglobulin (1 μg/mL) and the F1-peptide (concentrations shown on x-axis) were added to T2 cells and incubated for 18 h at 26 °C in a 5% CO_2_ incubator. Mean fluorescence intensity (MFI) was determined for the F1-peptide concentrations using flow cytometry; (**B**) MUC1 peptides (anchor-optimized and glycosylated) competed effectively with the F1-peptide. T2 cells were incubated with the reference peptide (F1-peptide, 100 ng/mL), β2-microglobulin (1 μg/mL) and increasing amounts of unlabeled MUC1 peptides (competitor peptides). The concentrations that produced 50% inhibition of the F1-peptide by the competitor peptides (IC_50_) were calculated as follows: (1 − (MFI T2 + F1-peptide + modified peptide − MFI T2 only)/(MFI T2 + F1-peptide − (MFI T2 only)) × 100. The software program Prism was used. Peptides were arbitrarily scored as low affinity binding peptides with IC_50_ of more than 15 μg/mL, medium affinity binding peptides with IC_50_ of more than/equal to 5 μg/mL and less than/equal to 15 μg/mL, and high affinity binding peptides with IC_50_ of less than 5 μg/mL.

Binding affinities were determined using a competitive peptide inhibition assay. Titration of the Hepatitis B core peptide F1 showed that the appropriate dynamic range and saturation were reached at ~150 ng/mL of peptide concentration (Figure 2A). The glycosylated and anchor-optimized peptides showed increased affinity of HLA-A*0201 compared to P1:STAPPVHNV or P5:LLLLTVLTV on T2 cells (Figure 2B). The IC_50_ for the two previously described peptides, P1:STAPPVHNV and P5:LLLLTVLTV, were 10.13 and 10.89 μg/mL, respectively (Figure 2B); hence, these two peptides fall within the IC_50_ range for “medium binders” [40]. The glycosylated or anchor-optimized peptides all showed high binding affinity, with IC_50_ ranging from 0.34 to 1.68 μg/mL. Optimization of leucine at position two (P15:SLAPPVHNV) in the P1:STAPPVHNV peptide and/or changing the proline in position five to a threonine (P3:SLAPTVHNV or P16:STAPTVHNV) resulted in a 22 to 30-fold increase in the binding affinity (Figure 2B). Adding a Tn residue to the threonine in the fifth position (P2:STAPT(Tn)VHNV or P4:SLAPT(Tn)VHNV) resulted in 6- to 8-fold increased binding compared to the native P1:STAPPVHNV peptide (Figure 2B). The degenerate tandem repeat peptide P9:ALGSTAPPV and its glycosylated counterpart both showed high-affinity binding as did the cytoplasmic tail peptide P7:SLSYTNPAV. IC_50_ values for the “high affinity binders” were comparable to the previously described positive control peptides from CEA (P12:YLSGADLNL) and CMV (P14:NLVPMVATV) [41]. Thus, all the modified peptides can be considered “high-affinity binders” which may serve well for developing MUC1-specific CTLs.

### 2.4. In Vitro Stimulation of T Cells from Normal HLA-A*0201 Women Elicited Strong MUC1-Specific CTL Responses

**Figure 3:**
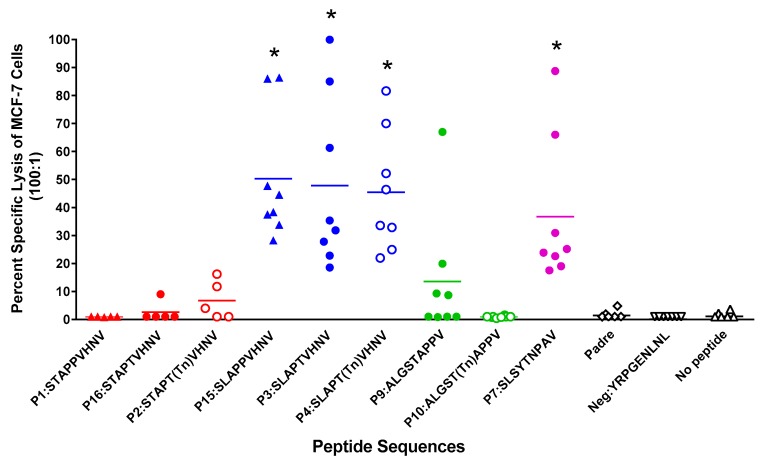
In vitro stimulation of T cells from normal post-menopausal HLA-A*0201^+^ women with anchor-optimized or glycosylated MUC1 peptides elicited strong CTL activity. PBLs underwent two rounds of stimulation and sorted CD8^+^ T cells were subjected to a ^51^Cr-release assay. Data represent killing activity of various MUC1-specific CTLs generated against MCF-7 (MUC1^+^, HLA-A2^+^) cells used as targets. Effector:target ratio was 100:1 and spontaneous release was less than 15% of complete lysis. * *p* = 0.008 compared to the negative peptide.

CTLs, generated from 8 normal post-menopausal HLA-A*0201 women stimulated in vitro with autologous DCs pulsed with glycosylated and/or anchor-optimized MUC1 peptides, elicited lysis of MCF-7 cells (MUC1^+^, HLA-A*0201^+^) in a ^51^Cr release assay (Figure 3). There was no CTL activity against the MDA-MB-231 cells (MUC1^−ve^, HLA-A*0201^+^) (data not shown). Peptides most effective at inducing significant lysis were those with leucine in position two (P15:SLAPPVHNV; P3:SLAPTVHNV; P4:SLAPT(Tn)VHNV; and P7:SLSYTNPAV, *p* = 0.008 for all, compared to the negative control peptide, P11:YRPGENLNL).

**Figure 4:**
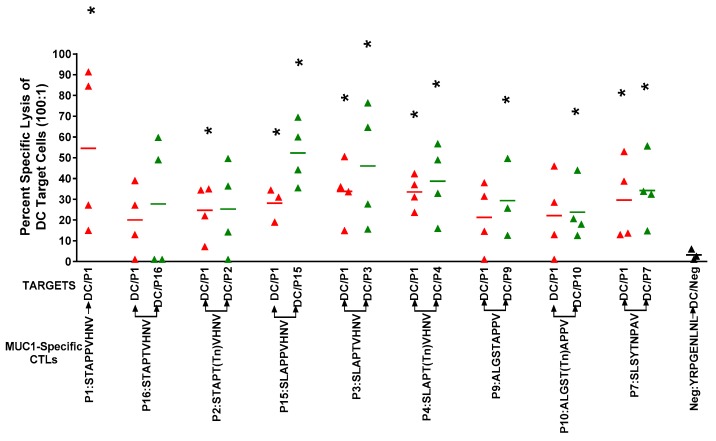
CTLs were lytic to DCs pulsed with the immunizing peptide and showed cross-reactive lytic activity to the native P1 peptide STAPPVHNV. Autologous DCs pulsed with various MUC1 peptides (50 μg/mL) and PADRE peptide (10 μg/mL) were used as targets. Effector:target ratio was 100:1 and spontaneous release was less than 15% of complete lysis. * *p* < 0.05 (Student’s *t*-test) compared to the negative peptide.

CTLs from four donors were also tested for reactivity to the immunizing peptide and cross-reactivity to the native peptide, P1:STAPPVHNV (Figure 4). Interestingly, CTLs were lytic against DCs pulsed with the native P1:STAPPVHNV peptide, which was not seen when MCF-7 cells were used as targets (Figure 3 and Figure 4). The disparities in lytic reactivity against MCF-7 and peptide-pulsed DCs as targets may be due to disparate glycosylation of the endogenously expressed MUC1 by MCF-7 cells. Furthermore, CTLs elicited by all peptides reacted against autologous DCs pulsed with the immunizing peptide or with the native peptide, P1:STAPPVHNV. Due to insufficient numbers of CTLs, we did not test cross-reactivity to the other peptides. We have previously shown in preclinical mouse studies that immunizations with either non-glycosylated or glycosylated peptides resulted in MUC1-specific T cells that recognized both naked and glycosylated antigens and intramolecular epitope spreading between the cytoplasmic tail and tandem repeat peptides [34]. The cross-reactivity between the native peptide P1 was very encouraging since we were unable to generate CTLs reactive against MCF-7 cells from the native peptide (P1:STAPPVHNV), which has been used in clinical trials previously and against which naturally occurring CTLs have been detected in breast cancer patients [42].

**Figure 5:**
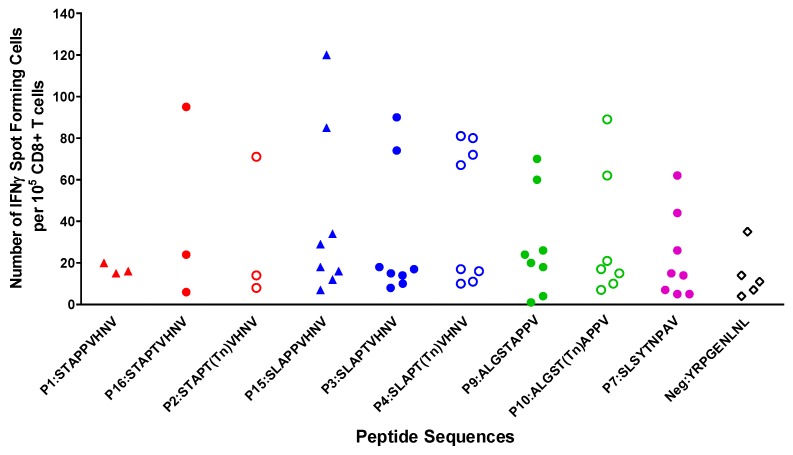
Production of IFNγ by CD8^+^ T cells was induced in response to MUC1 peptides. Following two rounds of stimulation, CD8^+^ cells were maintained for 24 h on an ELISpot plate. Spot numbers were determined using computer assisted video image analysis by Zellnet Consulting Inc. There were no significance differences compared to the negative peptide (*p* > 0.05).

ELISpot analysis of CD8^+^ T cells generated to MUC1 peptides optimized in the second position to leucine (P15:SLAPPVHNV) and/or in the fifth position to threonine or glycosylated threonine (P4:SLAPT(Tn)VHNV and P2:STAPT(Tn)VHNV) showed production of IFNγ (Figure 5). It should be noted that the IFNγ data did not always follow the same robust response as the CTL data. For instance, P3:SLAPTVHNV and P7:SLSYTNPAV elicited strong CTL responses but the same T cells showed low IFNγ production, whereas the CTLs from one individual elicited by P16:STAPTVHNV and P2:STAPT(Tn)VHNV showed strong production of IFNγ and low lysis (Figure 3 and Figure 5). We and others have previously noted that IFNγ production is not always predictive of CTL effectiveness [34,43,44].

### 2.5. Breast Cancer Patients Recognize and Proliferate to the MUC1 Peptides in Vitro

**Figure 6:**
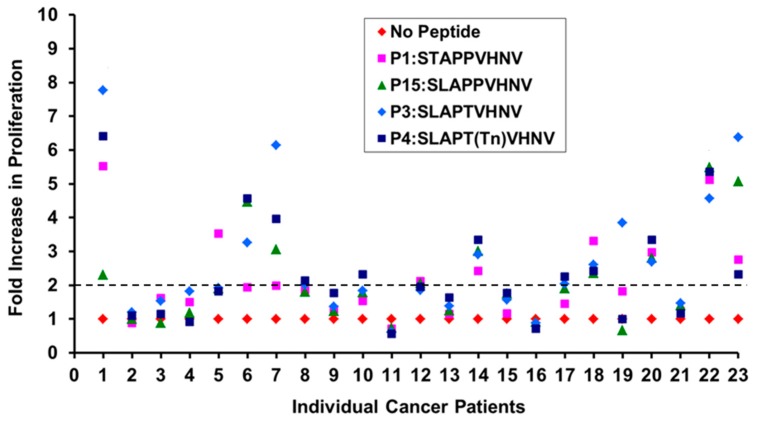
T cells from ~38% of the HLA-A2^+^ breast cancer patients regardless of their stage, ER/PR and HER2 status responded to the selected MUC1 peptides. 1 × 10^5^ T cells were incubated with irradiated allogeneic DCs pulsed with the various MUC1 peptides (10 μg/mL) + IL-2 for 5 days in complete media. Proliferation was assessed by measuring the ^3^H-thymidine uptake and is reported as fold increase in counts per minute. The dotted line indicates the two-fold increase in proliferation.

To determine if breast cancer patients have T cell repertoires that recognize these MUC1 peptides, we screened 23 HLA-A*0201 breast cancer patients regardless of their stage, ER/PR and HER2 status with four selected peptides (P1, P3, P4, P15). CD8^+^ T cells from the patients were incubated with irradiated autologous DCs pulsed with the various MUC1 peptides (10 μg/mL) plus IL-2 for 5 days and proliferation was assessed by measuring the ^3^H-thymidine uptake. T cells from ~38% of the breast cancer patients responded to the selected MUC1 peptides (Figure 6).

Considering >2-fold increase in proliferation as a positive response, compared to no-peptide stimulated T cells, 8/23 patients responded to P1:STAPPVHNV and P15 :SLAPPVHNV; 9/23 responded to P3:STAPTVHNV and 10/23 responded to P4:STAPT(Tn)VHNV (Figure 6). This led us to further investigate if the CD8^+^ T cells from breast cancer patients possessed the ability to become cytolytic against MUC1-expressing HLA-A*0201 breast cancer cells.

### 2.6. In Vitro Stimulation of T Cells from Breast Cancer Patients Elicited a Strong CTL Response

**Figure 7:**
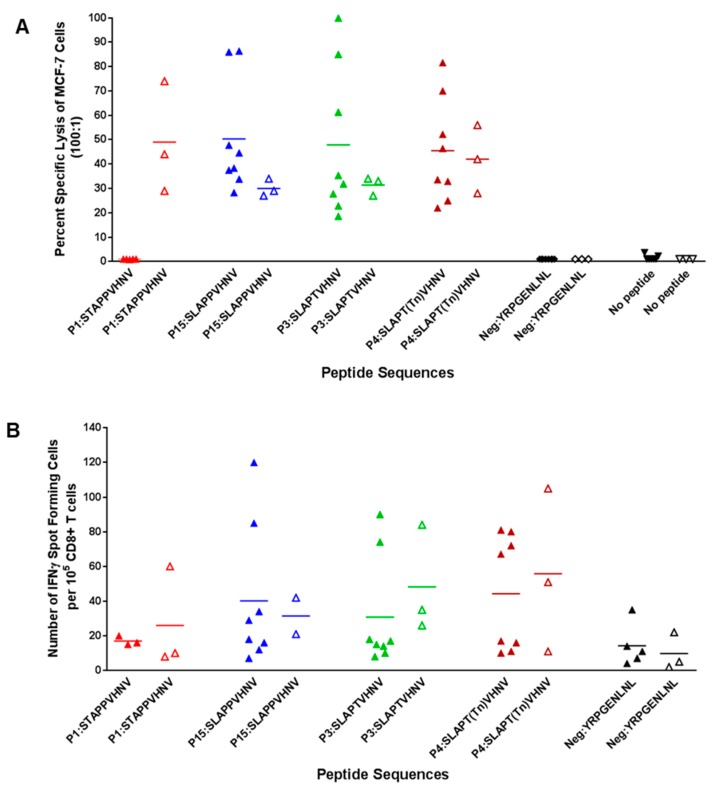
In vitro stimulation of T cells from HLA-A*0201^+^ cancer patients (peri-menopausal) with anchor-optimized and/or glycosylated MUC1 peptides elicited strong CTL activity. (**A**) PBLs underwent two rounds of stimulation and sorted CD8^+^ T cells were subjected to a ^51^Cr-release assay. Targets were MCF-7 cells (HLA-A*0201^+^, MUC1^+^). Effector:target ratio was 100:1 and spontaneous release was less than 25% of complete lysis. The filled triangles designate normal individuals and the open triangles designate breast cancer patients. For the peptides P3 and P4, there was no evidence that % specific lysis of MCF-7 cells in response to the peptide differed between the healthy donors and those with cancer (rank sum test; *p* > 0.05), although for the P1 peptide, there was evidence that the response to the peptide was greater in healthy donors than in those with cancer (Wilcoxon rank sum test; *p* = 0.04); (**B**) Production of IFNγ by CD8^+^ T cells was induced in response to MUC1 peptides. Following two rounds of stimulation, CD8^+^ cells were maintained for 24 h on an ELISpot plate. Spot numbers were determined using computer assisted video image analysis by Zellnet Consulting Inc. (FortLee, NJ, USA). There was no evidence of a significance difference in spot numbers between cancer patients and healthy controls (Wilcoxon rank sum test; *p* > 0.05).

To confirm that HLA-A*0201 breast cancer patients have a T cell repertoire similar to normal donors, three peptides (P3:SLAPTVHNV, P4:SLAPT(Tn)VHNV, and P15:SLAPPVHNV), which had elicited strong lytic responses from normal donors (Figure 3), were used for stimulation of T cells from three patients. The native peptide, P1, was included because T cells from one-third of breast cancer patients proliferated to it (Figure 6). All four of the peptides were equally good at stimulating lytic CD8^+^ T cells from the breast cancer patients against MCF-7 cells that express MUC1 endogenously (Figure 7A). Of note, the native peptide P1:STAPPVHNV, which did not elicit lytic T cells from normal donors (Figure 3), effectively activated lytic T cells from all three breast cancer patients. Induced T cells failed to lyse MDA-MB-231 cells that lack MUC1 but are HLA-A*0201^+^ (data not shown); this strongly suggests antigen-specific killing. T cells from the breast cancer patients showed IFNγ production in ELISpot analysis (Figure 7B), similar to levels observed in normal donors (Figure 5).

## 3. Discussion

We have identified two novel MHC class I peptides (an aberrantly glycosylated anchor-optimized heteroclitic peptide (P4:S**L**AP**T(Tn)**VHNV) and non-glycosylated heteroclitic counterparts (P15:S**L**APPVHNV and P3:S**L**AP**T**VHNV) that bind to HLA-A*0201 molecules with high affinity (8- to 30-fold higher affinity, respectively, than the known M1.1 and M1.2 MUC1 peptides) (Figure 2), resulting in a strong cellular immune response (Figure 3). These peptides elicited robust lytic CTLs from normal donors (Figure 3), as well as breast cancer patients (Figure 7) that were effective in killing MCF-7 breast cancer cells (HLA-A*0201^+^, MUC1^+^) at high efficiency (Figure 3 and Figure 7). Each peptide elicited lytic responses in six out of eight normal individuals. Based on considering 30% cell kill a response, it appears that at least 47% of donors receiving P15:SLAPPVHNV will respond and at least 35% of donors receiving P4:SLAPT(Tn)VHNV will respond (Table 2). This may, however, be different for cancer patients since T cells from all three cancer samples showed greater than 30% lysis of the MCF-7 cells when stimulated with P1, P3, P4, or P15. We also determined that the native peptide P1:STAPPVHNV was not immunogenic for normal T cells in vitro (based on the MCF-7 cell line) but was highly immunogenic for breast cancer T cells and that the lytic activity did not always correlate with the IFNγ production (Figure 3, Figure 5 and Figure 7A). The clinical significance of this data is not yet implicit but it certainly points to the cancer specificity of the native MUC1 peptide and perhaps suggests immune tolerance to native MUC1 in the normal individuals. It is indeed plausible that the MUC1-tolerance is circumvented in breast cancer patients which now express a different T cell repertoire than normal healthy age-matched women. Previous studies have demonstrated that T cells become activated due to the increased antigen load that results from tumor development [45]. More patient samples from various stages of the disease need to be tested before the relevance of the data can be fully understood.

We have shown recently that CTLs generated to glycopeptide antigens were protective against MUC1-expressing mouse tumors in MUC1 transgenic mice and that both glycosylated 9-mer peptides as well as non-glycosylated 9-mer peptides could induce both CD4^+^ and CD8^+^ T cells that produced IFNγ and recognized various glycosylated MUC1 peptides and tumor-associated MUC1 as well as controlled tumor growth in MUC1 transgenic mice with repetitive vaccination [34]. A similar result was obtained by Apostolopoulos, who generated CTLs against a MUC1 K^b^-binding peptide conjugated to Tn [46]. Glycosylation of the threonine (T) in the 5th position of the K^b^ binding peptide SAPDTRPA resulted in CTLs that recognized both the glycosylated and non-glycosylated MUC1 peptide. Modeling and crystal structure suggested that position 5 Thr-Tn carbohydrate is anchored in the central C pocket of the MHC binding cleft, thus increasing the binding affinity of the peptide for the class I molecule [46]. Binding affinity of the MUC1 peptides with a glycosylated Thr in position 5 and MHC class I was measured and found to be high, similar to the well-known H-2K^b^ OVA peptide, SIINFEKL. Addition of the Tn increased affinity 60-fold at 23 °C and 100-fold at 37 °C versus the non-glycosylated peptide; it stabilized H-2K^b^ molecules on RMA-S cells ~100-fold more efficiently than the non-glycosylated peptide [46]. Our data shows a similar pattern with increased affinity to MHC class I (Figure 1 and Figure 2) compared to the native peptide. The CTLs generated to the glycosylated-anchor optimized P4:SLAPT(Tn)VHNV also reacted against the native P1:STAPPVHNV (Figure 4). Our data and that of Apostolopoulos suggest that peptides bearing a single sugar on threonine or serine are likely to elicit CD8^+^ T cells with dual specificity, able to bind to glycosylated or non-glycosylated peptides, both heteroclitic or native to MUC1 [34,46].

The carbohydrate we selected for the vaccine is Tn, which consists of N-acetyl galactosamine, the first sugar to be *O*-linked on mucin proteins. This form of glycosylation is widely expressed in cancers, in particular by most breast cancers [14]. It appears early in the oncogenic process and is considered to be a neoantigen, suggesting that people will not have developed central tolerance to it.

Full consideration of the immune suppressive effects induced by tumors and the microenvironment is important, as many vaccine studies have been unsuccessful because of failure to address immune suppression [47]. Many chemotherapeutic drugs are involved in remodeling the tumor immuno-suppressive environment; it is well established that vaccines can be effectively combined with chemotherapeutics in a way that allows the vaccine to be more effective [48]. The recent successes of checkpoint inhibitors, specifically anti-PD1 and anti-CTLA4 with others under development, have prompted us to turn our attention to effective vaccine strategies, which induce tumor-specific T cell activation. These inhibitors sustain T cell activation (anti-PD1) and promote expansion of T cells by inhibiting T regulatory cells (anti-CTLA4), thus promoting response and immunity in distinct ways [49].

The potential therapeutic advantage implicit in our data is that the glycosylated and non-glycosylated heteroclitic peptides will bind to class I molecules more strongly and are likely to generate a strong CTL and clinical response [50]. The CTLs induced by these glycosylated and heteroclitic peptides reacted against the naturally glycosylated MUC1 on human breast tumor cells, suggesting that these analog peptides may be significantly better at inducing immune responses than the native antigen and could offer substantial improvements in the design of epitope-based vaccines, thus fulfilling MUC1’s potential as a therapeutic target. As MUC1 is a widely expressed tumor antigen, found on about 75% of tumors that kill, effective vaccine strategies with optimal peptides will have widespread applicability, especially as combined appropriately with immunomodulatory therapies such as checkpoint inhibitors.

## 4. Materials and Methods

### 4.1. Peptide Synthesis

Various HLA-A*0201 restricted MUC1 peptides were synthesized with modifications that enhanced binding (Table 1). The unmodified native MUC1 peptides used were P1 (STAPPVHNV, M1.1 [39]), P9 (ALGSTAPPV [51]) and P5 (LLLLTVLTV, M1.2 [39]). The MUC1 peptides were optimized at the second anchor position to leucine and/or at the fifth position to threonine or glycosylated threonine. P11:YRPGENLNL was used as the negative control and the positive controls used were P12 (CAP1-6D CEA: YLSGADLNL), P13 (EBV: GLCTLVAML) and P14 (CMV: NLVPMVATV) [24]. All non-glycosylated MUC1 peptides (except for P5:LLLLTVLTV), control peptides, and the peptide PADRE containing the HLA-DR binding epitope were synthesized at the Mayo Proteomics Research Center. P5:LLLLTVLTV was purchased from American Peptide Company, Inc. (Sunnyvale, CA, USA) with peptide purity of 42%. The glycosylated MUC1 peptides (P2:STAP**T(Tn)**VHNV; P4:S**L**AP**T(Tn)**VHNV and P10:ALGST**(Tn)**APPV) were synthesized using Fmoc chemistry on a MilliGen 9050 Synthesizer (Applied Biosystems, Foster City, CA, USA) at Arizona State University Protein Core facility. Tn-modifications were introduced at the fifth amino acid position of the peptide by using Fmoc-Thr(GalNAc(Ac_3_)-α-D)-OH (Bachem Bioscience, King of Prussia, PA, USA). Peptides were purified on the Beckman System Gold HPLC using a Jupiter Proteo C12 column (Phenomenex, Torrance, CA, USA) and an acetonitrile gradient. Peptides were greater than 95% pure as determined by mass spectrometry. The peptides were dissolved in phosphate-buffered saline (pH 7.4) to give 10 mg/mL stock solutions, aliquoted and stored at −70 °C. The F1-peptide (Hepatitis B core antigen_18–27_), which is an HLA-A*0201-binding peptide (FLPSDFFPSV), was synthesized with a cysteine residue substituted for the F (FLPSDCFPSV). This cysteine was conjugated to fluorescein (F1-peptide) for use in competitive inhibition studies to measure the affinity of peptides for HLA-A*0201 molecules (kind gift from Dr. Douglas Lake, Arizona State University, Tempe, AZ, USA).

### 4.2. MHC Stabilization Assay

Peptide binding to HLA-A*0201 was analyzed using TAP1- and TAP2-deficient T2 cells which express the HLA-A*0201 allele. T2 cells (2 × 10^5^) were added to increasing amounts of the MUC1 peptides (0–100 μg/mL) and β2-microglobulin (1 μg/mL) in a total volume of 100 μL AIM V medium per well in a round-bottom 96-well plate and incubated for 18 h at 37 °C. Binding of the peptides was measured using flow cytometry for upregulation of HLA-A2 surface expression on the T2 cells. The peptide-pulsed T2 cells were washed and stained with the FITC-labeled HLA-A2 antibody (clone BB7.2, Pharmingen, San Diego, CA, USA) prior to analysis. Mean fluorescence intensity (MFI) was determined and the fluorescence index (FI) was calculated from the formula: FI = (F_S_ − F_B_)/(F_T2_ − F_B_) × 100 where F_S_ is the MFI of the test peptides, F_B_ is the no-peptide isotype antibody-stained control MFI, and F_T2_ is the no-peptide HLA-A2 antibody-stained control MFI [52].

### 4.3. Competitive Peptide Inhibition Assay

In order to measure the affinity of the peptides for HLA-A2 molecules, competitive peptide inhibition assays were performed. The reference peptide, FLPSDCFPSV (F1) that was conjugated to fluorescein at the cysteine residue, was titrated to determine the concentration to be used in the competitive peptide inhibition assay. Recombinant β2-microglobulin (1 μg/mL) and various concentrations of the reference peptide were added to the T2 cells (2.5 × 10^5^ cells/200 μL serum-free RPMI medium per well) and incubated for 18 h at 26 °C in a 5% CO_2_ incubator. The concentration of 100 ng/mL of the F1-peptide was selected for the peptide competition assays. Relative peptide affinity for HLA-A2 molecules was determined by incubating T2 cells (2.5 × 10^5^ cells/200 μL serum-free RPMI medium per well) with F1-peptide (100 ng/mL), β2-microglobulin (1 μg/mL) and increasing amounts of unlabeled MUC1 peptides (0–100 μg/mL). The concentrations that produced 50% inhibition of the F1-peptide by the competitor peptides (IC_50_) were calculated as follows: (1 − (MFI T2 + F1-peptide + modified peptide − MFI T2 only)/(MFI T2 + F1-peptide − (MFI T2 only)) × 100. The software program Prism was used. Peptides were arbitrarily scored as “low affinity binders” with IC_50_ of >15 μg/mL, “medium affinity binders” with IC_50_ of ≥to 5 μg/mL and <15 μg/mL, and “high affinity binders” with IC_50_ of <5 μg/mL [40].

### 4.4. Isolation of PBMCs and HLA Testing

PBMCs were obtained from heparinized whole blood or from the cellular residue in the leucoreduction system chambers (LRSCs) after platelet pheresis of normal healthy post-menopausal women [53]. PBMCs were isolated by Ficoll-Paque (Amersham Bioscience, Uppsala, Sweden) density gradient separation, washed three times in PBS, and used immediately for further assays or cryopreserved in heat-inactivated fetal bovine serum (FBS from Hy-Clone) containing 10% DMSO. PBMCs from HLA-A*0201 positive donors were used for generation of peptide-specific CTLs in vitro. The non-adherent lymphocytes were separated from adherent cells and used as T cells and the adherent cells were used as dendritic cells (DCs) or antigen presenting cells (APCs).

### 4.5. In Vitro Induction of Peptide-Specific CTLs

An optimized protocol for the generation of peptide-specific CTLs was developed that relied upon autologous DCs for stimulation. PBMCs (2 × 10^6^ cells/mL of AIM V medium) were transferred to a 24-well plate and incubated for 2 h at 37 °C. Non-adherent lymphocytes were washed off with PBS and the adherent cells were cultured in X-VIVO medium containing GM-CSF (1000 U/mL) and IL-4 (1000 U/mL) to generate DCs. On day four, the DCs were washed with PBS and pulsed for 2 h with the specific MUC1 peptide (50 μg/mL) (Table 1) and PADRE peptide (10 μg/mL) along with recombinant β2-microglobulin (3 μg/mL) in freshly prepared X-VIVO medium (1 mL per well) containing GM-CSF (1000 U/mL) and IL-4 (1000 U/mL). Lipopolysaccharide (LPS) at 1 μg/mL and tumor necrosis factor-α (TNFα) at 10 ng/mL were added to the peptide-pulsed DCs for 48 h to mature the DCs. DCs were analyzed by flow cytometry for appropriate maturation markers. For the first stimulation (R1 stimulation), PBMCs (2 × 10^6^ cells/mL of T cell medium per well) were transferred to the irradiated and washed peptide-pulsed mature DCs at a stimulator to responder ratio of 1:10 in the presence of IL-7 (10 ng/mL). T cell medium was prepared by mixing equal volumes of EHAA (Click’s medium, Sigma-Aldrich, St. Louis, MO, USA) and RPMI 1640 with 1% GlutaMAX™, 100 U/mL penicillin, 100 μg/mL streptomycin, 100 μM 2-mercaptoethanol, and 10% heat inactivated human AB serum (Valley Biomedical Inc., Winchester, VA, USA). After three days, IL-2 at 1 ng/mL was added and refreshed every three days. On day 12 post R1 stimulation, CD8^+^ T cells were sorted using CD8 microbeads (Miltenyi, Sunnyvale, CA, USA) as per the manufacturer’s protocol. The sorted CD8^+^ T cells were rested for two days, and re-stimulated (R2 stimulation) with the peptide-pulsed mature DCs at a stimulator to responder ratio of 1:10 in the presence of IL-7 (10 ng/mL) and IL-2 (1 ng/mL). Five to seven days later, T cells were analyzed for cytolytic activity by ^51^Cr-release assay and interferon gamma production by ELISPOT.

### 4.6. Measurement of Cytolytic Activity of CTLs by ^51^Cr-Release Assay

The lytic activity of the in vitro stimulated peptide-specific CTLs was measured by a standard six-hour ^51^Cr-release assay. The target cells used were MCF-7 breast cancer cell lines which express MUC1^+^ and HLA-A*0201, and MDA-MB-231 cells, which are MUC1 negative and express HLA-A*0201. All tumor targets were treated with IFNγ one-day prior to use to up-regulate class I surface expression. Autologous DCs pulsed with the recall peptides, other modified peptides and the native peptide were also used as targets to evaluate cross-reactivity of MUC1-specific CTLs. The targets were labeled with Na^51^CrO_4_ (0.1 mCi/mL) for 2 h, washed 3 times with PBS and re-suspended in T cell medium to a concentration of 2 × 10^4^ cells/mL. Labeled target cells (2 × 10^3^ cells) were co-cultured with CTLs (2 × 10^5^ cells) at a ratio of one target to 100 effector cells in a total volume of 200 μL T cell medium in 96-well round-bottom plates for 6 h at 37 °C in a 5% CO_2_ incubator. Target cells were also treated with 5% Triton X-100 or medium only for complete and spontaneous ^51^Cr release, respectively. After incubation, 30 μL of supernatant was transferred onto LUMA plates (Perkin Elmer, Waltham, MA, USA) and radioactive ^51^Cr release was measured in a Topcount Micro-scintillation Counter (Packard Biosciences, Shelton, CT, USA). Percent specific lysis was calculated according to the following formula: Percent specific lysis = (experimental cpms − spontaneous cpms)/(complete cpms − spontaneous cpms) × 100. Spontaneous ^51^Cr release was less than 15% of complete ^51^Cr release.

### 4.7. Detection of IFNγ Secreting MUC1-Specific Cytotoxic CD8^+^ T Cells by ELISpot

IFNγ ELISpot plates from Mabtech (Stockholm, Sweden) were used to detect IFNγ producing MUC1-specific CTLs. The assay was performed using the capture IFNγ antibody as recommended by the manufacturer. Day five post R2-stimulation, the CTLs were washed three times and 1 × 10^5^ cells were transferred to the washed and blocked IFNγ ELISpot plates and incubated at 37 °C in a 5% CO_2_ incubator for 24 h. After incubation the plates were washed with PBS, incubated with alkaline phosphatase-conjugated IFNγ antibody at room temperature for two hours and then spots were detected with BCIP/NBT substrate. Spot numbers were determined using computer assisted video image analysis by Zellnet Consulting Inc. (Fort Lee, NJ, USA).

### 4.8. Ethics Statement

All subjects gave their informed consent for inclusion before they participated in the study. The study was conducted in accordance with the Helsinki Agreement, and the protocol was approved by the Institutional Review Board (IRB) of the Mayo Clinic on 7 February 2006 (IRB 06-002076). Normal volunteers (HLA-A2^+^), de-identified, were between the ages of 50 and 70 and could not have a history of cancer, autoimmune disease or take any immunosuppressive drugs. Breast cancer patients (HLA-A2^+^) between the ages of 50 and 70 were de-identified and recruited regardless of the tumor stage, ER/PR or HER2 status.

### 4.9. Statistics

Either two sample *t*-tests or Wilcoxon rank sum tests were used to assess differences among groups. SAS 9.3 (Cary, NC, USA) was used for analysis.

## 5. Conclusions

There remains great interest in the development of immunotherapy directed to antigens shared across tumors, which simplifies the manufacture and decreases the cost for production of an off-the-shelf vaccine for wide-spread use. MUC1 is a ubiquitously expressed protein found at high levels on the majority of tumors that kill. Our optimization of MUC1 peptides for activation of human T cells resulted in specific CTLs from normal donors and breast cancer patients that were highly effective in killing MUC1-expressing breast cancer cells. These analog peptides, heteroclitic and glycosylated with tumor-specific sugars, may offer substantial improvement in design of epitope-based vaccines. Vaccines, combined with checkpoint inhibitors that diminish tumor immunosuppression, are likely to generate an effective non-toxic anti-cancer response and/or complete eradication of cancer in patients.

## Figures and Tables

**Table 1:** List of MUC1 and control peptides for modification.

Human Peptide	Sequence	Gene	Modification
P:1	STAPPVHNV	MUC1	Degenerate TR
P:16	STAP**T**VHNV	MUC1	Modified
P: 2	STAP**T(Tn)**VHNV	MUC1	Glycosylated
P:15	S**L**APPVHNV	MUC1	Anchor-optimized
P: 3	S**L**AP**T**VHNV	MUC1	Anchor-optimized
P: 4	S**L**AP**T(Tn)**VHNV	MUC1	Glycosylated
P: 9	ALGSTAPPV	MUC1	Degenerate TR
P:10	ALGST**(Tn)**APPV	MUC1	Glycosylated
P: 7	SLSYTNPAV	MUC1	Cytoplasmic tail
P: 5	LLLLTVLTV	MUC1	Signal peptide
P:11	YRPGENLNL		None (Negative control)
P:12	YLSGADLNL	CEA	None (Positive control)
P:13	GLCTLVAML	EBV	None (Positive control)
P:14	NLVPMVATV	CMV	None (Positive control)

All peptides have been synthesized using Fmoc chemistry on a MilliGen 9050 Synthesizer (PerSeptive Biosystems). Tn modifications were introduced at the fifth amino acid position of the peptide by using Fmoc-Thr(GalNAc(Ac_3_)-α-D)-OH, which was purchased from Bachem Bioscience. Peptides were purified on the Beckman System Gold HPLC using a Jupiter Proteo C12 column (Phenomenex) and an acetonitrile gradient. Peptides were greater than 95% pure as determined by mass spectrometry. Modifications are noted in bold.

**Table 2:** Predicted percent of donors that will respond to MUC1-peptide immunization.

Peptide Sequence	% of Donors with at Least 30% Cell Kill	95% CI
S**L**APPVHNV	87.50%	47.3%–99.7%
S**L**AP**T(Tn)**VHNV	75%	34.9%–96.8%
S**L**AP**T**VHNV	62.50%	24.5%–91.48%
SLSYTNPAV	37.50%	8.5%–75.5%
ALGSTAPPV	12.50%	0.32%–52.6%

Estimation of the true proportion of donors who respond to the peptide and the corresponding 95% confidence interval: Based on considering 30% cell kill a response, it appears that at least 47% of donors receiving P15:SLAPPVHNV will respond and at least 35% of donors receiving P4:SLAPT(Tn)VHNV will respond.
